# Prophylactic Percutaneous Kyphoplasty Treatment for Nonfractured Vertebral Bodies in Thoracolumbar for Osteoporotic Patients

**DOI:** 10.1155/2020/8593516

**Published:** 2020-04-09

**Authors:** Fei Lei, Wen He, Xinggui Tian, Zhongyang Li, Lipeng Zheng, Jianping Kang, Daxiong Feng

**Affiliations:** ^1^Department of Spine Surgery, The Affiliated Hospital of Southwest Medical University, No. 25 Taiping St., Luzhou, 646000 Sichuan, China; ^2^Department of Library, Southwest Medical University, No. 1 Xianglin Road of Longma District, Luzhou, 646000 Sichuan, China; ^3^Department of Orthopedics Surgery, West China Hospital, Sichuan University, No. 37 Guoxue St. of Wuhou District, Chengdu, 610041 Sichuan, China

## Abstract

**Purpose:**

The occurrence of new vertebral compression fractures (VCFs) is a common complication after percutaneous kyphoplasty (PKP). Secondary VCFs after PKP occur predominantly in the thoracolumbar segment (T11 to L2). Prophylactic injections of cement into vertebral bodies in order to reduce new VCFs have rarely been reported. The main purpose of this study was to investigate whether prophylactically injecting cement into a nonfractured vertebral body at the thoracolumbar level (T11-L2) could reduce the occurrence of new VCFs.

**Methods:**

From July 2011 to July 2018, PKP was performed in 86 consecutive patients with osteoporotic vertebral compression fractures (OVCFs) in the thoracolumbar region (T11-L2). All patients selected underwent PKP because of existing OVCFs (nonprophylactic group). Additionally, 78 consecutive patients with fractured vertebrae in the thoracolumbar region (T11-L2) with OVCFs underwent PKP and received prophylactic injections of cement into their nonfractured vertebrae in the thoracolumbar region (T11-L2) (prophylactic group). The visual analog scale (VAS) scores and incidence of new VCFs after PKP were compared between the two groups.

**Results:**

The mean VAS scores improved from 8.00 ± 0.79 preoperatively to 1.62 ± 0.56 at the last follow-up in the nonprophylactic group and improved from 8.17 ± 0.84 to 1.76 ± 0.34 in the prophylactic group (*P* > 0.05). In the nonprophylactic group, 21 of 86 patients (24.4%) developed new VCFs within one year after PKP, of whom 15 patients (71.4%) developed VCFs within 3 months. In the prophylactic group, 8 of 78 patients (10.3%) developed new VCFs within one year, and 6 of these 8 patients (75%) developed new VCFs within 3 months. The incidence of new VCFs was significantly higher in the nonprophylactic group than that in the prophylactic group at one year (*P* = 0.018), but there were no statistically significant differences at three months (*P* = 0.847).

**Conclusions:**

Prophylactic injections of cement into nonfractured (T11-L2) vertebral bodies reduced the incidence of secondary VCFs after PKP in patients with OVCFs, but there was no significant difference in local back pain (VAS) scores between the two groups.

## 1. Introduction

Percutaneous kyphoplasty (PKP) is a minimally invasive vertebral augmentation technique that includes the injection of polymethylmethacrylate (PMMA) into fractured osteoporotic bodies, which can relieve local back pain quickly and reduce the number of complications due to long-term bedrest [1–4]. These techniques are widely used to treat patients with osteoporosis vertebral compression fractures (OVCFs), metastatic tumors, and multiple myeloma [5–8]. However, these techniques may be associated with several complications, such as an elevated risk for new vertebral compression fractures (VCFs), cement leakage, pulmonary embolism, and spinal cord or nerve injuries [9–13]. New VCFs after PKP result in severe local back pain, which often requires hospitalization and imposes a psychological burden on patients as well as an economic burden on society [14–17]. However, only a few studies have been reported [18–23] focusing on prophylactically injecting cement into adjacent vertebral bodies to reduce the number of new VCFs. Rho et al. [10, 15, 24] reported that secondary VCFs after PKP were mainly concentrated in the thoracolumbar region (T11 to L2). We investigated whether prophylactic injections of cement into nonfractured vertebral body in the thoracolumbar region (T11-L2) could reduce the occurrence of new VCFs.

## 2. Materials and Methods

### 2.1. Patients and Design

We retrospectively analyzed 164 patients between July 2011 and July 2018 and divided them into a nonprophylactic group and a prophylactic group. This study protocol was approved by the ethics committee of our hospital. All participants signed an informed consent form before they were included in the study. A total of 86 patients with OVCFs in the thoracolumbar region (T11-L2) who underwent PKP (nonprophylactic group) completed the follow-up. The inclusion criteria were as follows: (1) pain unresponsive to analgesics (visual analog scale (VAS) score≧5 points), (2) X-ray showing a VCF (a compression fracture with a minimum height loss of 15%), (3) magnetic resonance imaging (MRI) showing acute fractures (less than 3 weeks) as a hypointense signal on T1-weighted images and hyperintense signal on fat-suppressed sequences (STIR) images, (4) two or more fractured vertebral bodies located between T11 and L2 with a bone mineral density (BMD)≧2.5, (5) follow-up of more than one year, and (6) no pathological fractures.

Additionally, a total of 78 patients who completed the follow-up had OVCFs located in the thoracolumbar (T11-L2), underwent PKP (prophylactic group), and received prophylactic injections of cement into the nonfractured vertebral bodies in the thoracolumbar region (T11-L2). The inclusion criteria were as follows: (1) pain unresponsive to analgesics (VAS score≧5 points), (2) X-ray showing a VCF (a compression fracture with a minimum height loss of 15%), (3) MRI showing acute fractures (less than 3 weeks) as a hypointense signal on T1-weighted images and hyperintense signal on fat suppressed sequences (STIR) images, (4) two or more fractured vertebral bodies located between T11 and L2 with a BMD ≧2.5, (5) follow-up of more than one year, and (6) no pathological fractures.

### 2.2. Surgical Procedures

All procedures were performed by a senior surgeon with 10 years of experience in PKP. The patients were placed in a prone position and received local anesthesia (1% lidocaine); their blood pressure, electrocardiogram, and oxygen saturation levels were routinely monitored. An 11G bone marrow biopsy needle (Incheon SI Medical, Korea) was used to puncture the fractured vertebral body through the left or right pedicle, and the needle was inserted in the anterior third of the vertebral body under the guidance of C-arm fluoroscopy (Ziehm, Solo). Next, the needle was exchanged for a working cannula (SI Medical, Korea); a balloon (SI Medical, Korea) was placed into the vertebral body through the working cannula to expand the collapsed vertebral body. Polymethylmethacrylate (PMMA) (SI Medical, Korea) cement was injected into the vertebral body with 2 mL syringes. All patients underwent antiosteoporosis treatment, which included exercises, diet, alfacalcidol, zoledronic acid (5 mg intravenous injection once per year), and pain-relieving treatments (nonsteroidal anti-inflammatory drugs) postoperatively and were encouraged to ambulate as soon as possible after surgery.

### 2.3. Data Collection

Comparisons of VAS scores between the two groups were performed preoperatively and 1 day and 12 months postoperatively. Anterior-posterior and lateral radiographs were routinely obtained at 1 day and 1, 3, and 12 months postoperatively. A comparison of new fracture occurrence was performed between the two groups. If patients complained of local back pain after PKP, then MRI was necessary. If the involved vertebral body showed a hypointense signal on T1-weighted images and hyperintense signal on STIR images, then the occurrence of secondary VCFs was indicated.

### 2.4. Statistical Analysis

SPSS 21.0 software was used to perform the statistical analyses. Data are presented as the mean ± SD. Student's *t* test was used to compare continuous variables such as VAS scores. The chi-square test was used to compare dichotomous values (gender, incidence of new VCFs). The level of statistical significance was set at *P* < 0.05.

## 3. Results

All patients successfully underwent PKP intervention without severe complications (cement leakage into the spinal canal leading to spinal cord or nerve injuries, pulmonary embolism) during the perioperative period. The average amount of cement injected into each vertebral body was 2.5 to 3.5 mL in the middle to upper thoracic region, 3.0 to 4.5 mL in the thoracolumbar region, and 4.5 to 6.0 mL in the lower lumbar spine. The one-year follow-up was completed in all 86 cases in the nonprophylactic group and 78 cases in the prophylactic group (excluding 11 cases lost to follow-up). The demographic and baseline characteristics of both patients' groups are summarized in Table 1. No significant differences were observed between the two groups regarding patient age (*P* = 0.921), gender (*P* = 0.648), or BMD (*P* = 0.571).

**Figure 1:**
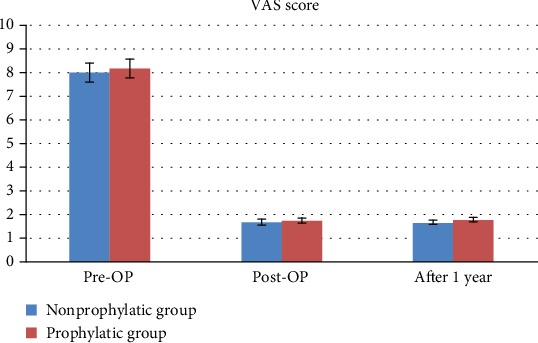
The mean VAS scores at preoperatively, postoperatively, and one year after surgery.

Pain was obviously reduced after the PKP intervention in both groups. The mean VAS scores decreased dramatically from a baseline (preoperative) value of approximately 8 to 1.5 immediately post-PKP in both groups. After one year, the VAS scores were slightly lower, but no significant difference was found between the two groups (1.62 ± 0.56 vs. 1.76 ± 0.34, *P* = 0.053; Table 2, Figure 1).

**Figure 2:**
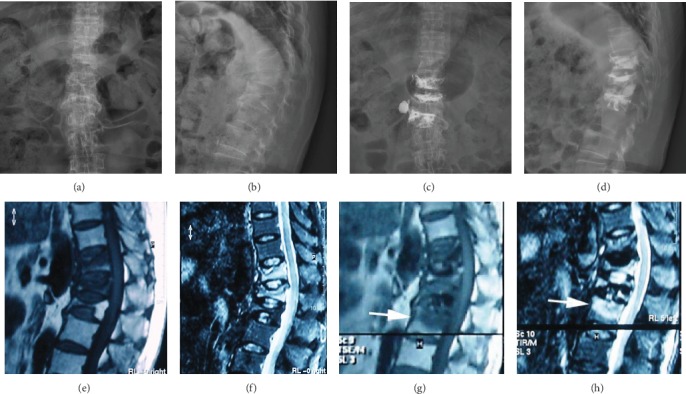
Preoperative X-ray of a 69-year-old woman with OVCFs of the T11, 12, and L1 vertebral bodies (a, b). MRI (T1-weighted and STIR-weighted images) visualized each fractured vertebral body (e, f). The BMD values were -3.5 (T11), -3.3 (T12), and -3.6 (L1). The patient underwent PKP from T11 to L1 (c, d). Seventeen days after surgery, the patient complained of low back pain, and MRI showed a new vertebral body fracture in L2 (white arrow) (g, h). The patient elected to receive conservative treatment.

**Figure 3:**
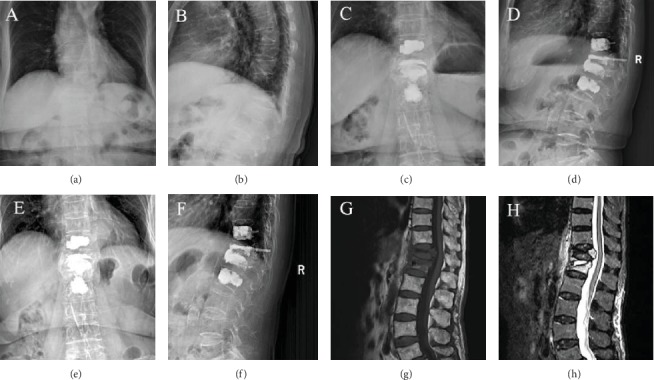
Preoperative X-ray of a 76-year-old woman with OVCF of the T12 and L1 vertebral bodies (a, b). MRI (T1-weighted and STIR-weighted images) visualized each fractured vertebral body (g, h). The BMD values were -3.9 (T12) and -3.4 (L1). The patient underwent PKP from T12 to L1, and T11 and L2 were treated with prophylactic PKP. X-rays were obtained after PKP (c, d). Twelve months after PKP, no evidence of new VCFs was observed (e, f).

In the nonprophylactic group, 59.3% (51/86) of the initially OVCFs occurred between T11 and L2, 24.4% (21/86) of the patients had new VCFs, of which 71.4% (15/21) of the fractures occurred within 3 months after the intervention, and only 6 fractures occurred after 3 months (Figure 2). Of these new VCFs, 76.2% (16/21) were adjacent vertebral body fractures, only 23.8% (5/21) were remote vertebral body fractures, and 9.5% (2/21) were recollapse of the cemented vertebra. 61.9% (13/21) of new VCFs occurred between T11 and L2. In the prophylactic group, 10.3% (8/78) of the patients had new VCFs, of which 75% (6/8) occurred within 3 months after surgery, and 2 cases occurred after 3 months. Adjacent vertebral body fractures occurred in 62.5% (5/8) of the cases, remote vertebral body fractures occurred in 3 cases 37.5% (3/8), and recollapse of the cemented vertebra occurred in 25% (2/8) of the cases. The incidence of new VCFs was significantly different between the two groups (Table 2, Figure 3, *P* = 0.018). However, the incidence of new VCFs within 3 months after PKP (*P* = 0.847) and the incidences of adjacent vertebral fractures, remote vertebral fractures, and recollapse of the cemented vertebrae were not significantly different between groups (Table 2, *P* = 0.461, *P* = 0.300, and *P* = 0.646, respectively).

## 4. Discussion

PKP using PMMA is widely used to achieve swift local back pain relief in patients with OVCFs [5–8]. New VCFs after PKP are a well-known phenomenon, during follow-up due to the osteoporosis disease, and are not necessarily related to the vertebral augmentation technique [25–29]. Due to differences in statistical methods, inclusion criteria, experimental designs, and follow-up times, different incidences of subsequent vertebral body fractures after PKP have been previously reported. In the literature, [30–33] reported the incidence of new vertebral fractures ranges from 8% to 52% after PKP. In our study, the incidence of new VCFs in the nonprophylactic group was 24.4% (21/86) after PKP during the one-year follow-up and was 10.3% (8/78) in the prophylactic group. The difference between the two groups was statistically significant (*P* = 0.018). The corresponding incidences during the first three months were 71.4% (15/21) and 75% (6/8), with no statistically significant difference between groups (*P* = 0.847). Therefore, the first year after PKP, particularly the first three months, is the key period for new VCFs to occur.

The thoracolumbar segment is a sensitive area because of its unique anatomical structure, which can predispose this region to fracture development following trauma. Voormolen et al. [34] reported that 70.6% (72/102) of the initially OVCFs were concentrated in the thoracolumbar segment (T10 to L2). The authors also concluded that more than two preexisting VCFs were an independent risk factor for the development of new VCFs. Rho et al. [10] reported a refracture rate of 70.4% (19/27) of refracture after PKP was performed in the thoracolumbar segment (T11 to L2). In our study, in the nonprophylactic group, 59.3% (51/86) of the initially OVCFs occurred between T11 and L2, 61.9% (13/21) of the new VCFs occurred in the thoracolumbar segment. Kobayashi et al. [20] observed some effects of prophylactic percutaneous vertebroplasty (PVP) treatment with OVCFs. The authors performed prophylactic PVP in the nonfractured vertebral body adjacent to the fractured vertebrae of 155 patients, with no prophylactic PVP in a control group of 89 patients. The incidence of new VCFs was 4.5% and 16.8% during the three months, and the new fractures were mainly adjacent to the vertebral fractures; the one-year incidence was 9.7% and 22.4% in the prophylactic and control groups, respectively. However, in this study, cement was injected only cranially or caudally around the fractured vertebral body, without considering interventions for other vertebral bodies with risk factors, such as more than two preexisting vertebral body fractures located in the thoracolumbar region (T11-L2). In the prophylactic group of our study, PKP was performed for fractured vertebrae in thoracolumbar (T11-L2), and cement was prophylactically injected into nonfractured vertebral in the thoracolumbar (T11-L2) region of the same patient. The incidence of new VCFs in the prophylactic group was obviously lower than that in the nonprophylactic group (10.3% versus 24.4%, *P* = 0.018) after PKP during the one-year follow-up. Thus, we believe that prophylactic injections of cement into nonfractured thoracolumbar region (T11-L2) vertebral bodies may reduce the occurrence of new VCFs after PKP.

New VCFs after PKP intervention included those that affected the adjacent vertebral bodies, recompression of cemented vertebral bodies, and remote vertebral body fractures. After PKP, the load-bearing kinetics redistribute to other vertebrae, especially those adjacent to the original fracture, which increases the risk for adjacent vertebral body fractures. Takahara et al. [35] reported that the incidence of adjacent vertebral body fractures was 94.7% (18/19) after PKP, while Rho et al. [10] reported that it was 66.7% (18/27). In our study, the incidence of adjacent vertebral fractures was 76.2% (16/21) in the nonprophylactic group and 62.5% (5/8) in the prophylactic group, with no statistically significant difference between groups (*P* = 0.461). In total, 23.8% (5/21) of remote vertebral body fractures occurred in the nonprophylactic group, and 37.5% (3/8) occurred in the prophylactic group, which was not a statistically significant difference (*P* = 0.300).

Recollapse of the cemented vertebral body after PKP intervention frequently occurs, and previous studies [14, 36–38] have reported different risk factors for this complication after PKP intervention for OVCFs, such as preoperative intravertebral cleft, preoperative severe kyphosis, fracture level concentrated in the thoracolumbar region, high vertebral height restoration, and poor cement distribution. Kim and Rhyu [36] stated that the recompression rate for cemented vertebrae was 12.5%. Chen et al. [39] retrospectively analyzed 134 patients with OVCFs who underwent PKP and found that 9.7% of the patients developed recollapse of the cemented vertebral body. In our study, the incidence was 9.5% (2/21) in the nonprophylactic group and 25% (2/8) in the prophylactic group, with no statistically significant difference between groups (*P* = 0.646).

Lindsay et al. [26] reported patients with OVCFs treated with conservative management. These patients exhibited a 20% incidence of new VCFs during one year of follow-up, and in patients with more than two vertebral body fractures preoperatively, the incidence increased to 24%. The incidence of new VCFs after conservative treatment was similar to that after PKP, as described previously [10, 40], because vertebral body fractures are part of the natural progression of osteoporosis [41] rather than being directly related to PKP. Therefore, PKP does not change the natural course of osteoporosis. Kamano et al. [18] reported that patients who underwent prophylactic PKP still experienced new VCFs. This study also confirms that PKP cannot alter underlying osteoporosis. Becker et al. [23, 42] stated that there is no basis for prophylactic PKP treatment for osteoporotic VCFs. Therefore, it is still controversial whether prophylactic PKP treatment should be performed, and further studies are necessary to validate this approach.

Our study had several limitations. First, this was a single-center retrospective study with a small number of cases and a midterm follow-up. Second, we did not include information about new VCFs treated conservatively. Consequently, the incidence rate of new VCFs after PKP intervention was lower than the true rate. Third, prophylactic PKP increased radiation exposure to the patients and surgeons during the surgical procedure, prolonged the surgery time, and increased the risk of surgery.

## 5. Conclusions

Prophylactic injections of cement into nonfractured thoracolumbar vertebral bodies reduced the incidence of additional secondary VCFs after PKP in patients with OVCFs, but there was no significant difference in pain relief between groups.

## Figures and Tables

**Table 1:** The demographic and baseline characteristics of patients in two groups.

	Nonprophylactic group	Prophylactic group	Statistic test	*P*
Number of patients	86	78	*—*	—
Age (years)	71.2 ± 7.8	70.4 ± 8.3	*t* = 0.099	0.921
Gender (M/F)	27/59	21/57	*χ* ^2^ = 0.209	0.648
BMD (T-score)	−2.93 ± 0.42	−3.02 ± 0.53	*t* = 0.568	0.571

^∗^ *P*<0.05. Data are mean ± SD; BMD: bone mineral density.

**Table 2:** Clinical status of patients after procedure.

	Nonprophylactic group	Prophylactic group	Statistic test	*P*
VAS score pre-OP	8.00 ± 0.79	8.17 ± 0.84	*t* = −1.33	0.187
VAS score post-OP	1.68 ± 0.56	1.84 ± 0.49	*t* = −1.96	0.052
VAS score at 1 year	1.62 ± 0.56	1.76 ± 0.34	*t* = −1.95	0.053
New VCFs	24.4% (21/86)	10.3% (8/78)	*χ* ^2^ = 5.636	0.018^∗^
New VCFs within 3 months	71.4% (15/21)	75% (6/8)	*χ* ^2^ = 0.037	0.847
Adjacent new VCFs	76.2% (16/21)	62.5% (5/8)	*χ* ^2^ = 0.544	0.461
Remote new VCFs	23.8% (5/21)	37.5% (3/8)	—	0.300
Recollapse of cemented vertebrae	9.5% (2/21)	25% (2/8)	—	0.646

^∗^ *P* < 0.05. Data are mean ± SD; VCF: vertebral compression fractures.

## Data Availability

The datasets used and/or analyzed during the current study are available from the corresponding author on reasonable request.
